# The Diagnostic Value of Indocyanine Green in the Assessment of Depth of Burn Injuries: A Systematic Review

**DOI:** 10.3390/ebj7010019

**Published:** 2026-03-19

**Authors:** Marie K. Hilgarth, Samuel Knoedler, Gabriel Hundeshagen, Adriana C. Panayi, Bong-Sung Kim, Jochen-Frederick Hernekamp, Valentin F. M. Haug

**Affiliations:** 1 Medical Faculty, HMU Health and Medical University Potsdam, 14471 Potsdam, Germany; marie.hilgarth@student.hmu-potsdam.de; 2 Department of Plastic Surgery and Hand Surgery, Klinikum Rechts der Isar, Technical University of Munich, 80333 Munich, Germany; 3 Department of Hand, Plastic and Reconstructive Surgery, Microsurgery, Burn Trauma Center, BG Trauma Center Ludwigshafen, University of Heidelberg, 67071 Ludwigshafen, Germany; 4 Department of Oral and Maxillofacial Surgery, Charité—Universitätsmedizin Berlin, Corporate Member of Freie Universität Berlin, Humboldt Universität zu Berlin, 10117 Berlin, Germany; 5 Department of Plastic Surgery and Hand Surgery, University Hospital Zurich, 8091 Zurich, Switzerland; 6 Department of Plastic and Reconstructive Surgery, Vivantes Hospital Friedrichshain, 10249 Berlin, Germany

**Keywords:** burns, burn depth assessment, ICG, indocyanine green

## Abstract

Background: Accurate assessment of burn depth remains a clinical challenge and requires specific training. To improve diagnostic accuracy, various technical methods have been developed. This review summarizes current evidence on indocyanine green (ICG) fluorescence imaging for burn depth assessment and compares its performance with clinical, histological, and alternative modalities such as Laser Doppler imaging (LDI). Methods: A systematic literature search was conducted in PubMed/MEDLINE, Cochrane and Google Scholar to identify studies evaluating burn depth using ICG fluorescence imaging. Studies from 1995 to 2024 were included if they compared ICG to at least one reference method (clinical assessment, biopsy, or other technical modalities). Data extraction was performed independently by two reviewers. Risk of bias was assessed using the Newcastle–Ottawa Scale. The study selection workflow is shown in the PRISMA 2020 flow diagram for systematic reviews. Results: Nine studies with a total of 151 patients, published between 1995 and 2024, met the inclusion criteria. Results were synthesized descriptively due to substantial methodological heterogeneity. Two studies reported high accuracy of ICG fluorescence imaging for identifying nonviable tissue and supporting surgical planning, although differentiation between superficial and deep partial-thickness burns (SPTBs/DPTBs) was inconsistent. In one study, ICGA-guided assessment reduced or avoided excision in 10 of 20 burn sites (50%). Yet heterogeneity in measurement protocols, cut-off values, and reference standards limited comparability across studies. Conclusions: Due to its limited accuracy in differentiating SPTBs and DPTBs, ICG imaging has restricted utility for burn depth assessment, though it may still offer intraoperative benefit during necrosectomy. Registration: PROSPERO International prospective register of SRs by the National Institute of Health Research (CRD420251161190).

## 1. Introduction

The classification of burns into four stages is essential for guiding treatment, including fluid resuscitation, the need for surgery, and predicting patient outcomes. Particularly critical is the differentiation between superficial partial-thickness burns (SPTBs) and deep partial-thickness burns (DPTBs), as this determines whether conservative management is sufficient or surgical debridement with excision and grafting is required.

Accurate clinical estimation of burn depth, however, remains challenging even for experienced surgeons [1]. To improve diagnostic accuracy and aid in surgical decision-making, several techniques have been investigated, including biopsy and histological assessment, infrared thermography, and indocyanine green (ICG) fluorescence imaging. Miccio et al. [2] initially used infrared thermography in 2016 in a porcine model to determine whether temperature differences in burn wounds in the first three days could predict burn depth. LDI was first used in 1989 by Waxman et al. [3] to analyze the burn depth and therefore the likelihood of burn wound healing.

The second-window ICG (SWIG) technique is based on the administration of a higher intravenous dose of indocyanine green, typically several hours prior to imaging. Due to string plasma protein binding and increased vascular permeability in injured or neoplastic tissue, ICG may accumulate within areas of tissue damage and reman detectable by near-infrared imaging despite systemic clearance from the circulation [4,5]. This was first used in 2012 in a study by Madajewski et al. [5] to determine the accumulation of ICG in residual tumors 24 h after intravenous administration of 7.5 mg/kg following standard resection in murine flank tumors.

The initial human trial of ICG for estimating burn depths was performed in 1995 by Sheridan et al. [6] as explained below.

ICG is a non-toxic fluorescent vital dye widely used in medicine as an indicator substance for cardiovascular, liver, and eye diseases. Intraoperative use in flap perfusion assessment facilitates the visualization of hypoperfused areas at an early stage [7]. This has been demonstrated to have a significant impact on reducing the overall failure rate and increasing the salvage rate of flap surgeries [8]. Following intravenous injection, imaging can be performed immediately after 20–30 s [9]. While initially limited to static images, technological advances now allow video-based visualization of ICG uptake, distribution, and clearance, enabling real-time assessment of tissue perfusion [4,10]. The blood flow in healthy and undamaged tissue is measured as a reference [11].

After intravenous administration, ICG reaches the liver by passing the bloodstream, and is excreted unchanged and almost completely (97%) into the bile in a non-conjugated form [12]. Through biliary excretion, the unconjugated metabolites are delivered to bile ducts and eliminated by the intestine. Small amounts can also be excreted by the kidneys.

Unmodified ICG undergoes a rapid metabolization process and therefore does not remain in the body for long, supporting its widespread use in medical diagnostic imaging. In a recently published scoping review, none of the 19 included studies involving a total of 324 patients (250 kidney transplant recipients and 74 with chronic kidney disease) reported any ICG-related adverse events or deterioration in renal function [13].

Nevertheless, as with any intravenously administered contrast agent, potential risks must be considered. Although renal toxicity appears unlikely, hypersensitivity reactions—including anaphylaxis, urticaria and procedural hypotension—have been reported, with patients undergoing hemodialysis potentially at increased risk. Careful patient selection and monitoring are therefore warranted [13,14].

Despite these considerations, ICG angiography (ICGA) is increasingly being used and serves several purposes in clinical practice: in burns, ICG fluorescence imaging provides real-time feedback of the tissue perfusion and viability, theoretically allowing more accurate delineation of burn extent compared to clinical assessment and informing decisions such as early excision of non-viable tissue and grafting, thereby potentially reducing mortality [10,12,15].

If there is a need for surgical debridement, ICG fluorescence imaging can help identify vital and perfused tissue from non-viable tissue in areas of mixed depth of injury, thus minimizing unnecessary excision of healthy tissue and promoting wound healing [4]. In the further course of regeneration, ICG can be used for monitoring the perfusion and the healing progress of the burned area. Due to changes in fluorescence intensity, surgeons can evaluate the effectiveness of the treatment and adjust the therapy to support wound healing [16]. Quantitative perfusion assessment may further identify wounds at higher risk of complications, such as transformation to necrotic tissue due to inflammation and ischemia, facilitating timely intervention [4].

Interference with other diagnostic measures (e.g., bilirubin testing, blood gas analysis) as well as imaging artifacts caused by ointments or dressings may result in misinterpretation [17]. Thus, ICGA requires proper dosing, careful application by trained personnel, and standardized imaging protocols to ensure reliability.

This review aims to synthesize the available evidence on the diagnostic performance of ICG fluorescence imaging for burn depth assessment compared with reference methods, including biopsy, clinical assessment, and second-window ICG techniques.

## 2. Materials and Methods

This systematic review was registered a priori on the PROSPERO International prospective register of SRs by the National Institute of Health Research CRD420251161190. No amendments were made to the protocol after registration.

### 2.1. Data Sources

Two independent reviewers (V.H., M.H.) performed a data search by using PubMed/MEDLINE, Cochrane and Google Scholar. The databases were searched from November 1995, when the first human trial analyzing the accuracy of ICG was published, until November 2024.

### 2.2. Search Strategy

The electronic search strategy was developed before study selection and applied consistently across all databases. Boolean operators (AND, OR, NOT) were applied to refine the search. The following combination of free-text terms was used: (“indocyanine green” OR “ICG”) AND (“burn” OR “burn depth” OR “burn depth assessment” OR “burn depth determination”).

No filters regarding study design were initially applied to maximize sensitivity. The reference lists of all included articles were manually screened to identify additional eligible studies.

### 2.3. Study Selection

The study selection was performed independently by both reviewers in a two-step process. First, titles and abstracts were screened; if abstracts were unavailable, the full text was reviewed. Full texts were then assessed against predefined inclusion and exclusion criteria. Disagreements between the reviewers were resolved through discussion and consensus. Eligible data were extracted and compiled into an Excel spreadsheet.

The following data were extracted: first author, title, publication year, publishing journal, country of institution of lead author, study design, number of patients, age, sex, wound location, total body surface area (TBSA), length of follow up (LOFU), length of stay (LOS), body mass index (BMI), time of interventions after injury, co-risk factors (alcohol use, smoking, hypertension, dyslipidemia, diabetes), etiology of burn (flame, contact, electrical, scald, friction, chemical), mortality, method of assessment and method of comparison, measurement times, dose of ICG and cut-off point.

### 2.4. Inclusion

Retrospective and prospective studies, including clinical trials, randomized controlled trials, cohort studies, and case series, were eligible if they investigated burn patients and assessed burn depth using ICG:

- Reporting on the main measurement method, as well as on the comparison and control method;
- Reporting on the use of ICG to determine burn depth, regardless of TBSA, LOS, LOFU, age 0–100.

The literature search was limited to studies published in English and German.

### 2.5. Exclusion

Exclusion criteria were unpublished studies, cost-effectiveness studies, case reports (*n* < 3), studies not reporting original data (reviews, guidelines, book chapters, literature reviews), non-human, veterinarian and cadaver studies, pediatric studies, and studies which were not published in German or English.

### 2.6. Quality Assessment

The methodological quality of the studies included in the systematic review was assessed by two independent reviewers (M.H., V.H.) using the Newcastle–Ottawa Scale (NOS) [18].

## 3. Results

**Figure 1:**
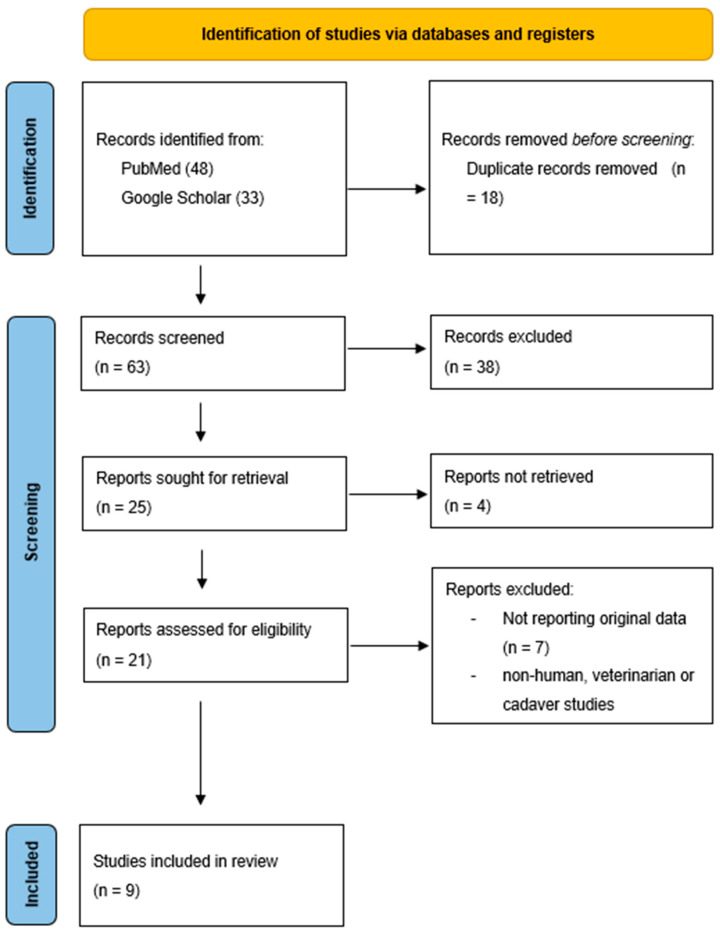
PRISMA flow chart for burn studies analyzing indocyanine green fluorescence imaging.

The study selection workflow is shown in the PRISMA 2020 flow diagram for systematic reviews (Figure 1). A total of 61 papers were screened for studies assessing burn depth using ICG, of which *n* = 18 were removed due to duplicates. Of these, 38 were excluded during title and abstract screening. Following the application of eligibility and exclusion criteria, a further 15 studies were eliminated. Nine studies, comprising 151 patients, were included in the systematic review. The records were published between 1995 and 2022 in the USA (*n* = 4), Thailand (*n* = 3), India (*n* = 1) and Austria (*n* = 1). Due to the limited number of studies with analyzable data and the substantial methodological heterogeneity among them, a quantitative synthesis in the sense of a meta-analysis was not possible. Instead, we present a descriptive overview of the available evidence (Table 1).

Sheridan et al. [6] conducted the first study on ICG for burn depth assessment, examining ten patients. Fluorescence images were captured at 825 mm and 750 mm excitation after intravenous injection of 0.2 mg/kg ICG. Measurements were performed at 5 min for five patients and at 1, 2, 3, 4, 5 and 10 min for the remaining five. The clearest differentiation between superficial and full-thickness burns, with the lowest standard deviation, was observed at 5 min. Images were compared with intraoperative assessment and clinical outcomes.

Dissanaike et al. [19] analyzed the potential of portable bedside ICG fluorescence measurement in determining the perfusion differences between areas of different burn depths and the likelihood of healing of patients with indeterminate burn depths and a TBSA < 15%, using an SPY machine in addition to standard burn care. After an intravenous dose of 5 mg ICG, perfusion images were screened within 1–2 min, followed by the washout process, which was completed after around 5 min. Using computerized perfusion analysis relative to baseline vital tissue, they demonstrated the potential of ICG to differentiate burn depths as early as day one.

In a prospective, multicentered, triple-blinded, experimental study, Wongkietkachron et al. [20] analyzed 30 burn sites with the aim of comparing the difference between ICGA and clinical assessment of the depth of indeterminate burn wounds and a biopsy with histopathological assessment as a control method. ICG demonstrated 100% accuracy, sensitivity, and specificity relative to histopathological results, indicating perfect agreement with the reference method and superior diagnostic performance compared with clinical assessment in distinguishing between superficial partial-thickness burns and non-superficial partial-thickness burns. The authors noted that melanin can absorb ICG wavelengths, potentially leading to false interpretations, and emphasized that ICGA should supplement, rather than replace, clinical judgement.

In a subsequent clinical trial, Wongkietkachron et al. [21] assessed 20 burn sites 2.3 days postinjury using a 33% maximal perfusion cut-off to differentiate superficial (≥33%) from deep (<33%) second-degree burns. Significant differences were observed between ICGA and clinical assessment, influencing surgical decisions: excision could have been omitted in four wounds and reduced in six, whereas in six cases ICGA indicated a larger excision area than suggested by clinical assessment. Overall, 19 of 20 sites achieved complete closure by day 21 (95%).

Korambayil et al. [22] analyzed ICG-infused burn wounds using monochrome video capture in 20 patients. In 13 patients, no difference was observed between clinical assessment and ICGA. The study concluded that ICG may improve burn outcomes and reduce unnecessary surgical interventions.

Zajac et al. [4] compared ICG imaging with SWIG imaging in mice, human skin xenografts and human burn models, with the conclusion of ICGA being a non-standardized and inconsistent modality for evaluating burn injuries. In contrast SWIG identified burn necrosis dependent on the dose and timing of ICG injection, with an inverse fluorescence signal compared to ICGA.

Wongkietkachorn et al. [23] further investigated short- and long-term outcomes after ICGA-guided excision in 30 burn sites, applying 33% maximal perfusion cut-off. Complete wound closure was achieved in 96.7% (29/30) in the short term and in 100% at two months, exceeding expected closure rates of 80%.

Kamolz et al. [10] examined ICGA in 20 patients to evaluate its utility in clinical decision making. Deep wounds appeared darker and more heterogenous in fluorescence images, and deeper burns correlated with longer ICG absorption, distribution, and elimination times. Histology analysis confirmed that reduced fluorescence and altered kinetic patterns corresponded to deep dermal vascular damage, while reference tissue imaging of normal skin serves as a perfusion baseline for comparison.

Still et al. [16] studied nine patients (15 burn sites, mean TBSA 23.11 ± 18.54) 4.79 ± 4.33 days post-burn. After intravenous injection of 0.1 mg/kg ICG and 785 nm near-infrared illumination, images were recorded for 5 min. SPTBs showed bright, diffuse fluorescence; DPTBs showed darker, mottled fluorescence; third-degree burns exhibited only large vessels with minimal fluorescence. Biopsy and clinical assessment served as references.

The quality assessment of the included studies using the Newcastle–Ottawa Scale (NOS) [18] is summarized in Table 2. Overall, the methodological quality varied among the studies. Most prospective studies showed moderate quality, with scores ranging between five and seven. The multicenter studies by Wongkietkachorn et al. [20,21,23] achieved the highest ratings.

**Table 1:** Summary of included studies.

Study	Design	Number of Patients (*n*)	Method of Assessment	Comparison Method	Key Findings	Limitations
Dissanaike et al. [19]	case series	3	ICG (SPY: portable device)	ICG fluorescence on unburned skin	- No significant difference in absolute perfusion in the cases requiring surgery and the cases that healed - Difficulty in defining a clear threshold for a surgery indication - Timing of the intervention significantly affects the results	- Small sample size - No long-term follow-up - Subjective interpretation of results of ICG measurement
Still et al. [16]	preliminary clinical trial	9	ICG	Biopsy, clinical assessment	- Agreement between ICG measurements, clinical assessment and histological evaluation	- Small sample size - No long-term follow-up - Variability in burn causes - Subjective image interpretation
Kamolz et al. [10]	prospective, case series	20	ICG	Biopsy, clinical assessment	- Agreement between ICG measurements and clinical assessment - Objective method to detect burn wound perfusion changes	- No long-term follow-up
Zajac et al. [4]	prospective	9	ICGA with SPY	SWIG fluorescence with NIR	- Precise detection of necrotic tissue in burn wounds with SWIG - Dependence on dose and timing - Inverse fluorescence signal compared to ICGA - Improved tissue margin identification	- Effectiveness of SWIG highly dependent on administered ICG dose and timing of injection - ICGA: inconsistent and not standardized, affecting comparability of results - Small sample size - No long-term follow-up
Sheridan et al. [6]	prospective	10	ICG	Clinical assessment	- Imaging conducted within five minutes post-ICG injection provided best contrast	- Small sample size - Included burns of different severities - No established protocols for conducting and interpreting fluorescence imaging - No long-term follow-up
Korambayil et al. [22]	prospective	20	ICG	Clinical assessment	- In 13/20 patients there were no differences between the two methods - ICG method improves outcome of burn wounds and can prevent surgical interventions by differentiating DPTBs to SPTBs	- No long-term follow-up
Wongkietkachorn et al. [20] (2019)	prospective, multicentered, triple-blinded	30	ICG	Biopsy, clinical assessment	- 100% accuracy of ICGA; 50% accuracy of clinical assessment - Clinical assessment: sensitivity 33.3%; specificity 66.7% - ICGA: sensitivity 100%; specificity 100% - NNT = 2	- No long-term follow-up - Involved only indeterminate burn wounds
Wongkietkachorn et al. [21] (2021)	prospective, multicentered, triple-blinded	20	ICG	Clinical assessment	- Precise wound marking, leading to 96.7% rate of complete wound healing - Improved diagnostic accuracy: ICGA 100%; clinical assessment 50% - Wounds marked by ICGA were on average 150% larger - Faster healing: >90% of burns identified as superficial by ICGA, which were clinically assessed as deep, healed within 21 days	- Variability in burn causes - No long-term follow-up
Wongkietkachorn et al. [23] (2021)	prospective, multicentered double-blinded	30	ICG	Clinical assessment	- High rate of complete wound closure: 96.7% short-term rate, 100% long-term rate - Early intervention with ICGA improves outcomes - ICGA provides objective, reliable burn depth differentiation, reducing unnecessary surgeries	- Restricted study population: involved only indeterminate burn wounds; easily classifiable burns were excluded - Not randomized - Short-follow up period (2 months) - Focus on diagnostic definition (superficial vs. deep burns) rather than therapeutic definition (need for excision or not) - Gray zone (25–45% perfusion)

**Table 2:** Quality assessment of included cohort studies using the Newcastle–Ottawa Scale (NOS) *. Studies with a NOS score of ≥7 are highlighted in bold.

Studies	Selection (max. 4 ★)	Comparability (max. 2 ★)	Assessment (max. 3 ★)
Dissanaike et al. [19]	NA	NA	NA
Still et al. [16]	NA	NA	NA
Kamolz et al. [10]	★★☆☆	★☆	★★☆
Zajac et al. [4]	★★★☆	★☆	★★☆
Sheridan et al. [6]	★★☆☆	NA	★★☆
Korambayil et al. [22]	★★☆☆	★☆	★★★
**Wongkietkachorn et al. (2019) [20]**	★★★★	★★	★★★
**Wongkietkachorn et al. (2021) [21]**	★★★★	★★	★★★
**Wongkietkachorn et al. (2021) [23]**	★★★★	★★	★★★

* Newcastle-Ottawa Scale (NOS). Good quality: 3 or 4 stars in selection domain AND 1 or 2 stars in comparibility domain AND 2 or 3 stars in outcome/exposure domain. Fair quality: 2 stars in selection domain AND 1 or 2 stars in comparibility domain AND 2 or 3 stars in outcome/expsure domain. Poor quality: 0 or 1 star in selection domain OR 0 stars in dombaribility domain OR 0 or 1 star in outcome/exposure domain.

## 4. Discussion

This systematic review provides a comprehensive overview of the current state of evidence regarding the use of indocyanine green fluorescence imaging for burn depth assessment. Although some studies report promising results, particularly in relation to surgical treatment planning, the overall validity and generalizability of the technique remain limited. Despite the systematic methodology applied, the available evidence could only be synthesized descriptively. This is primarily attributable to substantial methodological heterogeneity, the limited number of cases examined and the lack of standardized imaging and interpretation protocols.

The findings of this systematic review indicate that the ICG fluorescence technique does not represent a sufficiently reliable method for accurate burn depth determination, particularly regarding the differentiation between superficial and deep partial-thickness burns (SPTBs/DPBs). This distinction is of critical clinical relevance, as it directly influences subsequent treatment decisions. Although ICGA enables visualization of viable and non-viable tissue, its diagnostic performance appears limited to a coarse estimation of burn depth rather than precise stratification.

Accurately determining the depth of burn injuries requires sufficient experience and expertise and can be particularly challenging for inexperienced medical personnel. Even experienced physicians only achieve an accuracy of 60–75% in estimating the correct depth of burn injuries [1]. Since a correct estimation of the depth of burn injuries is crucial for the subsequent therapy of patients with burn injuries, a range of additional methods, such as ICGA, infrared thermography, or LDI, have repeatedly been tested alongside clinical assessment.

According to Sheridan et al. [6], the most reliable difference in burn depth was observed five minutes after ICG injection. Similarly, Dissanaike et al. [19] demonstrated that bedside ICGA measurements may allow accurate depth assessment as early as the first day following injury. In addition, Wongkietkachorn et al. [23] reported 100% sensitivity, specificity and overall accuracy, when ICGA findings were compared with biopsy and histopathological examination.

Nevertheless, the fact that ointments, bandages and blood reduce absorption and lead to the assumption of incorrectly deep wounds should not be neglected within this context. It is recommended to remove ointments, bandages and blood approximately ten minutes prior to the initial ICG measurement to ensure the attainment of reproducible and significant results [17].

Building on these results, and as further supported by Wongkietkachorn et al. [20] and Korambayil et al. [22], an ICG-guided approach may optimize surgical excision of burn wounds and facilitate a more precise evaluation of the necessity for operative treatment. Moreover, ICG-assisted debridement has been associated with high wound healing rates during follow-up.

In this context, a more pragmatic application of this technology can lie in the assessment of necrotic tissue and its use as an intraoperative adjunct during necrosectomies, where real-time perfusion imaging may help preserve viable tissue and potentially improve surgical outcomes.

As ICGA necessitates intravenous dye administration, it is inherently invasive and associated with catheter-related complications, systemic renal and hepatic burden, and rare allergic reactions, which may restrict its clinical utility in already compromised patients.

Owing to its non-invasive character and higher diagnostic precision, LDI appears to be superior to ICG imaging and may be a more reliable method for assessing burn depth according to Wang et al. [24]. LDI can be used to determine the burn depth and is based on the Doppler principle. Mono-frequency light waves are emitted and change their frequency as soon as they hit moving objects, such as blood cells. After performing this procedure, LDI devices can generate a color-coded perfusion map, based on a “flux” scale, which is equivalent to the varying burn depths and can be taken as proportional to the perfusion of the tissue [25].

In addition to its non-invasive application, this method can avoid infections and minimizes additional microtrauma and patient discomfort [26,27,28]. Previous studies have demonstrated associations with shorter hospital stays, fewer surgical interventions, more rapid decision-making regarding skin grafting, and reduced healthcare costs [26,27,28]. Nevertheless, broader validation through standardized multicenter trials remains necessary before routine implementation can be recommended.

Infrared thermography determines the temperature of burn wounds by measuring the thermal radiation of the tissue in the far-infrared range of the electromagnetic spectrum (wavelength 7–13 mm). As, in deeper burn injuries, a major amount of the vasculature is damaged, the hypoperfusion of the tissue and diminished perfusion lead to a subsequently reduced heat emission. The measured temperature can then be used as an indicator of the depth of the burn [29]. However, this method is limited by the existing loss of the surface barrier and the resulting heat loss, which can cause wounds to be interpreted as falsely deep. In addition, the measurement should be taken within three days of the event, as the onset of wound granulation influences the accuracy [30].

Overall, the use of laser-induced fluorescence by ICG in burn wounds may be able to provide real-time imaging that helps to make a precise determination of the burn depth, to guide through surgical interventions, to monitor the tissue perfusion and to make predictions about wound healing.

### Strengths and Limitations

The cohort size and thus the patient selection varied in some studies. In addition, different or unmentioned inclusion and exclusion criteria lead to limited comparability of the studies. The use of different control methods, imaging protocols and data interpretation to assess the ICG measurement method could induce inconsistencies in results.

There are also differences in the timing and intervals of measurement, which is relevant for determining the depth of combustion, as the dynamics of perfusion change in the early post-injury period, as demonstrated by Dissanaike et al. [19]. Without a standardized time point, measurements may reflect different stages of microvascular alteration or burn wound progression, potentially resulting in variable depth classifications.

In some studies, the assessment methods were not blinded, which could lead to biased interpretations (Table 1). Given the inherently subjective nature of clinical burn depth evaluation, the accuracy of ICG measurements could be influenced by the examiner’s level of expertise. Studies employing histopathological assessment as the reference standard were less inclined to such bias; however, variability in tissue processing and histological interpretation may still have impacted the results.

The included studies also predominantly report positive findings, raising the possibility of publication bias due to the potential underreporting of negative or inconclusive results. Furthermore, a considerable proportion of the evidence originates from the same research group (Wongkietkachorn et al. [20,21,23]), which may influence the diversity of methodological approaches and limit external validation.

To reduce bias, standardized protocols for ICG administration, imaging and interpretation should be established. García et al. [31] likewise demonstrated that a defined ICGA analysis workflow improves burn depth assessment and yields more reliable results, which also should be further validated in prospective multicenter trials.

## 5. Conclusions

ICG fluorescence imaging demonstrates potential as an adjunctive modality for intraoperative assessment of tissue viability and surgical decision-making in burn management. However, the currently available evidence does not support its use as a reliable primary tool for deep partial-thickness burns. At present, ICGA should be regarded as a complementary technique rather than a substitute for established diagnostic approaches. Further standardized, adequately powered multicenter studies are required to clearly define its role in clinical practice.

## Data Availability

No new data were created or analyzed in this study.

## Abbreviations

The following abbreviations are used in this manuscript:

ICG	Indocyanine Green
ICGA	Indocyanine Green Angiography
LDI	Laser Doppler Imaging
SWIG	Second-window Indocyanine Green
PRISMA	Preferred Reporting Items for Systematic Reviews and Meta-Analysis
DPTBs	Deep partial-thickness burns
SPTBs	Superficial partial-thickness burns
SWIG	Second-window ICG
TBSA	Total body surface area
LOFU	Length of follow up
LOS	Length of stay
BMI	Body mass index
NOS	Newcastle–Ottawa Scale
